# Review of Dr. Caldwell’s Pamphlet, Entitled Physiology Vindicated, in a Critique on Liebig’s Chemistry

**Published:** 1843-12-30

**Authors:** 


### BIBLIOGRAPHICAL NOTICES.

*Review of Dr. Caldwell's pamphlet, entitled Physiology Vindicated, in a Critique on Liebig's Chemistry.* By Robert Peter, M. D. Professor of Chemistry and Pharmacy, in Transylvania University. Cincinnati: 1843. 8vo. pp. 45.

This is an able refutation of the logical errors, misconceptions, and mis-statements of the *brochure* of Professor Caldwell. The author is evidently at home; he is master of his subject, and the fallacious reasoning, and unscientific character of this singular production are cleverly exposed. The present unstable condition of physiological chemistry, demands a most rigid scrutiny of every new doctrine that is advanced, no matter how plausible. But its claim should be candidly examined, and recognised facts, or constant and close analogies should be applied to test its correctness. Idle declamation, and angry vituperation of men of merit and science, will never destroy an unsound or vicious theory. The claims of the organic chemists have recently been extravagant; but a large portion of the agitation has been caused by individuals without the ranks, but who hoped to profit in the confusion and excitement. This fermentation has, we think, in a great measure subsided; and however actively the contest may be raging between the individual potentates, the great powers, *i. e.* the multitude, show but little interest, and are content to abide the results when announced.

The subjoined remarks on this subject, which we have recently met with, are so just that we shall transcribe them.

"The passing remarks which I have made on the new views of Liebig in physiological chemistry, may not be without their use; as they may serve to awaken inquiry, and to facilitate your future studies. Without doubt Liebig's physiological speculations are well worthy of your attention; but allow me to caution you against adopting them on trust, and without rigid examination. Let us be grateful to him and his coadjutors for the multitude of exact organic analyses which they have contributed; let us freely admit that they have shed much light upon particular points of physiology; but let us not at the same time be deceived into the belief that they have demonstrated the manner in which the nutrition and transformation of the tissues take place. It may be that they have lifted a corner of the curtain which conceals from our view what is performed on the stage of the vital organism; but let us be careful how we admit their presumptuous claim that they have penetrated behind the scenes, and witnessed nature in the very act of playing her part in the mysterious drama of life."*

* Introductory Lecture to the Course of Chemistry, in Jefferson Medical College, delivered Nov. 6th, 1843. By Franklin Bache, M. D.

